# Recent advances and emerging perspectives in vascular and cardiovascular research: A 2025 update

**DOI:** 10.1038/s41440-025-02540-1

**Published:** 2026-01-09

**Authors:** Shinji Kishimoto, Yukihito Higashi

**Affiliations:** 1 Department of Regenerative Medicine, Division of Radiation Medical Science, Research Institute for Radiation Biology and Medicine, Hiroshima University, Hiroshima, Japan; 2 Division of Regeneration and Medicine, Medical Center for Translational and Clinical Research, Hiroshima University Hospital, Hiroshima, Japan

**Keywords:** Atherosclerosis, Endothelial function, Arterial stiffness, Cardiovascular

## Abstract

Cardiovascular diseases (CVDs) have been a major cause of global morbidity and mortality, necessitating continuous innovation in diagnostic methods, better mechanistic understanding, and the development of risk stratification strategies. This review summarizes significant updates in vascular and cardiovascular health from 2024 to 2025, focusing on novel non-invasive assessment technologies, deeper insights into molecular and cellular pathophysiology, and effective approaches to clinical risk assessment. Key advancements include the development and validation of artificial intelligence-driven models for vascular age assessment, plethysmographic methods for endothelial function evaluation, and refined pulse wave velocity measurements for proximal aortic stiffness. Mechanistic studies have further investigated the roles of long noncoding RNAs, mitochondrial dynamics, and Piezo ion channels in various CVD pathologies. Clinically, new evidence supports the importance of central arterial stiffness in atrial myopathy, the association of pulse wave velocity with cerebral microbleeds, and the prognostic value of supine hypertension and combined vascular biomarkers, such as the cardio-ankle vascular index and ankle-brachial index. Furthermore, these updates will improve our understanding of vascular health and provide novel approaches to early detection, personalized intervention, and improving patient outcomes in the management of CVD.

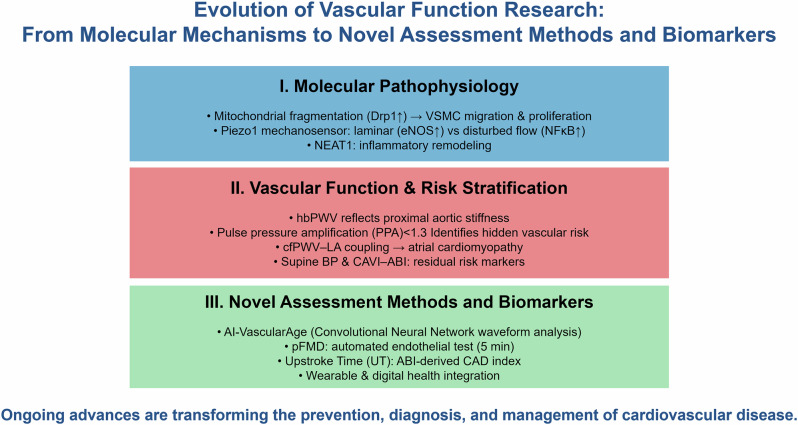

## Introduction

Vascular function and vascular stiffness play a critical role in maintaining overall cardiovascular health, and its impairment is indicative of various cardiometabolic disorders, leading to increased risk of adverse cardiovascular events [1]. The recent emergence of the concept of “vascular–organ interaction” has suggested that endothelial dysfunction and atherosclerosis represent a shared pathophysiological substrate that links the heart, kidneys, and brain [2, 3]. Impaired endothelial signaling reduces organ perfusion and promotes microvascular rarefaction, whereas stiff conduit arteries transmit excessive pulsatile energy to the microcirculation, thereby increasing hemodynamic load and contributing to peripheral organ dysfunction. Consequently, higher pulse wave velocity (PWV) has been consistently associated with damage to the brain (white matter lesions and cerebral small vessel disease) [4, 5], heart (left ventricular diastolic dysfunction and heart failure with preserved ejection fraction) [6, 7], and kidneys (reduced estimated glomerular filtration rate and microalbuminuria) [8]. Non-invasive vascular function tests have been increasingly recognized for their usefulness in assessing atherosclerosis severity, understanding disease pathophysiology, and assessing therapeutic interventions.

The period from 2024 to 2025 has seen a surge in research, providing novel insights and further knowledge in this dynamic field. This review aims to consolidate these recent developments, novel assessment technologies, complex molecular mechanisms, and their significant clinical implications for risk stratification and disease management and to review a list of articles on vascular or cardiovascular studies in Hypertension Research and other major cardiovascular and hypertension journals from 2024 to 2025 that were prepared by the editorial office of Hypertension Research.

## Emerging molecular mechanisms and pathophysiology

A better understanding of the molecular and cellular mechanisms underlying vascular dysfunction provides novel targets for therapeutic intervention.

**Fig. 1:**
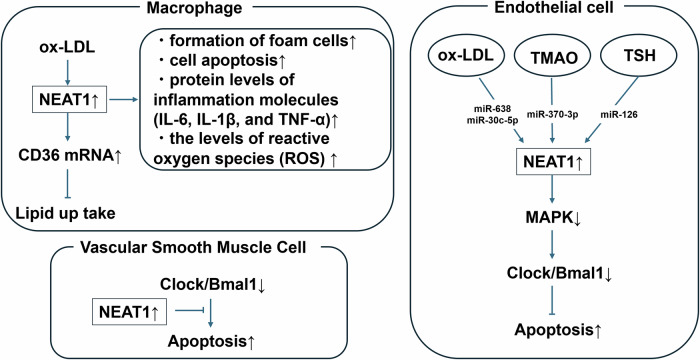
Cell type–specific mechanisms by which nuclear enriched abundant transcript 1 (NEAT1) contributes to atherosclerotic progression. In macrophages, exposure to oxidized low-density lipoprotein (ox-LDL) upregulates NEAT1, which increases CD36 mRNA expression and enhances lipid uptake. NEAT1 activation further promotes foam cell formation, macrophage apoptosis, and the production of inflammatory mediators (IL-6, IL-1β, and TNF-α), accompanied by high levels of reactive oxygen species (ROS). In vascular smooth muscle cells, NEAT1 suppresses the Clock/Bmal1 pathway and augments apoptosis. In endothelial cells, ox-LDL, trimethylamine N-oxide (TMAO), and thyroid-stimulating hormone (TSH) increase NEAT1 expression through distinct microRNAs (miR-638 and miR-30c-5p for ox-LDL, miR-370-3p for TMAO, and miR-126 for TSH). NEAT1 subsequently inhibits MAPK signaling, leading to the downregulation of the Clock/Bmal1 axis and an increased in endothelial apoptosis. Collectively, NEAT1 integrates metabolic, inflammatory, and endocrine cues to drive pro-atherogenic responses across multiple vascular cell types

Long noncoding RNAs (lncRNAs) are a class of non-coding RNAs over 200 nucleotides long, increasingly implicated in CVD pathogenesis [9]. LncRNA nuclear enriched abundant transcript 1 (NEAT1) has been established as a crucial factor in the pathological processes of various CVDs, including myocardial infarction, heart failure, myocardial ischemia-reperfusion injury, atherosclerosis, hypertension, cardiomyopathy, cardiac fibrosis, and aneurysm [10]. Increasing evidence has implicated the lncRNAs NEAT1 in atherosclerosis pathogenesis (Fig. 1). In macrophages, oxLDL induces NEAT1 expression via MAPK/NF-κB, limiting lipid uptake by sequestering CD36 mRNA while enhancing inflammatory cytokine release through miR-128 and miR-342-3p [11–13]. In endothelial cells, NEAT1 was upregulated by oxLDL, TNF-α, and TMAO, thereby promoting apoptosis, inflammation, and circadian disruption via the miR-638/PGK1, miR-30c-5p/TCF7, miR-126/TRAF7, and miR-370-3p/STAT3 pathways [14–16]. Conversely, in vascular smooth muscle cells (VSMCs), NEAT1 exhibited protective effects against apoptosis and circadian dysregulation [17]. Clinically, NEAT1 is increased in the serum and plaques of patients with atherosclerosis and coronary artery disease [18]. These findings indicated NEAT1 as a cell-specific regulator and a potential biomarker and therapeutic target in atherosclerosis. Although most studies have demonstrated NEAT1 upregulation in various cardiovascular diseases, a few studies have shown the opposite trend. These discrepancies may reflect differences in animal models, cell types, or racial and ethnic backgrounds of the study populations. Moreover, the current evidence base is derived largely from preclinical research, with limited validation in human clinical settings. Accordingly, well-designed and standardized experimental studies are necessary to resolve the inconsistency in the existing literature and to clarify the biological role of NEAT1 with greater reliability. Clinical investigations are also needed to determine whether NEAT1 can be translated into a viable therapeutic target and to establish optimal intervention strategies in humans.

**Fig. 2:**
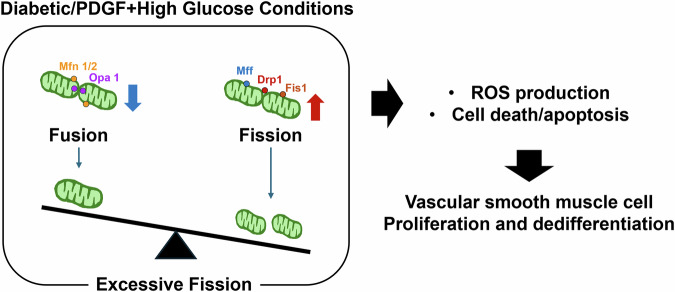
Mitochondrial division is mainly regulated by fission-related proteins, including dynamin-related protein-1 (Drp1), mitochondrial fission factor (Mff), and mitochondrial fission 1 protein (Fis1). Conversely, mitochondrial fusion is mediated by the GTPases mitofusin-1 (Mfn1), mitofusin-2 (Mfn2), and optic atrophy protein-1 (Opa1). The equilibrium between these opposing processes characterizes mitochondrial morphology and function, which in turn influences numerous cellular activities. When fission becomes excessive, mitochondria generate an overabundance of reactive oxygen species (ROS) and exhibit increased susceptibility to apoptosis and other types of cell death. These maladaptive responses contribute to the onset and progression of cardiovascular and vascular disorders

Mitochondria, beyond energy production, are crucial for various cellular functions, with their morphology regulated by active fusion and fission cycles [19, 20]. This balance between fusion and fission influences cell phenotype (Fig. 2). Excessive mitochondrial fission, which is offen mediated by dynamin-related protein 1 (DRP1), has been shown to CVD risk [21]. Intimal hyperplasia, a major cause of restenosis after vascular intervention, is commonly observed in patients with diabetes mellitus (DM) [22]. This pathology is critically driven by the phenotypic shift of the VSMCs and exacerbated by excessive mitochondrial fission [23]. DRP1 is established as a key mediator of mitochondrial fission, with its increased GTPase activity known to significantly worsen DM-induced intimal hyperplasia [24]. Zhang et al. have shown that isoliquiritigenin (ISL), a flavonoid, is a promising therapeutic agent [25]. Moreover, ISL tends to attenuate intimal hyperplasia by binding to Lys216 within the GTPase domain of DRP1, thereby reducing its GTPase activity and inhibiting mitochondrial fission [25]. This mechanism subsequently leads to a decrease in reactive oxygen species (ROS) generation, the suppression of VSMC proliferation and dedifferentiation, and ultimately, the alleviation of intimal hyperplasia in both in vivo diabetic mouse models and in vitro human aortic smooth muscle cells. These findings suggest that ISL provides a novel therapeutic strategy for diabetic vascular complications by precisely modulating DRP1-mediated mitochondrial dynamics. Nevertheless, the therapeutic targeting of DRP1 requires careful consideration, as both acute and chronic modulation may exhibit beneficial or detrimental effects. As most current evidence is derived from cultured cells and genetically modified animal models, it is necessary to extend these investigations to human samples [26]. The development of diagnostic and therapeutic strategies, including biopsy-based assessments and the identification of indirect biomarkers of mitochondrial dynamics, is important for clinical translation. Furthermore, quantitative and clinically applicable diagnostic and therapeutic tools for evaluating mitochondrial dynamics must be developed. This evaluation includes not only direct assessment via tissue biopsy but also the identification of indirect biomarkers reflecting these dynamics.

**Fig. 3:**
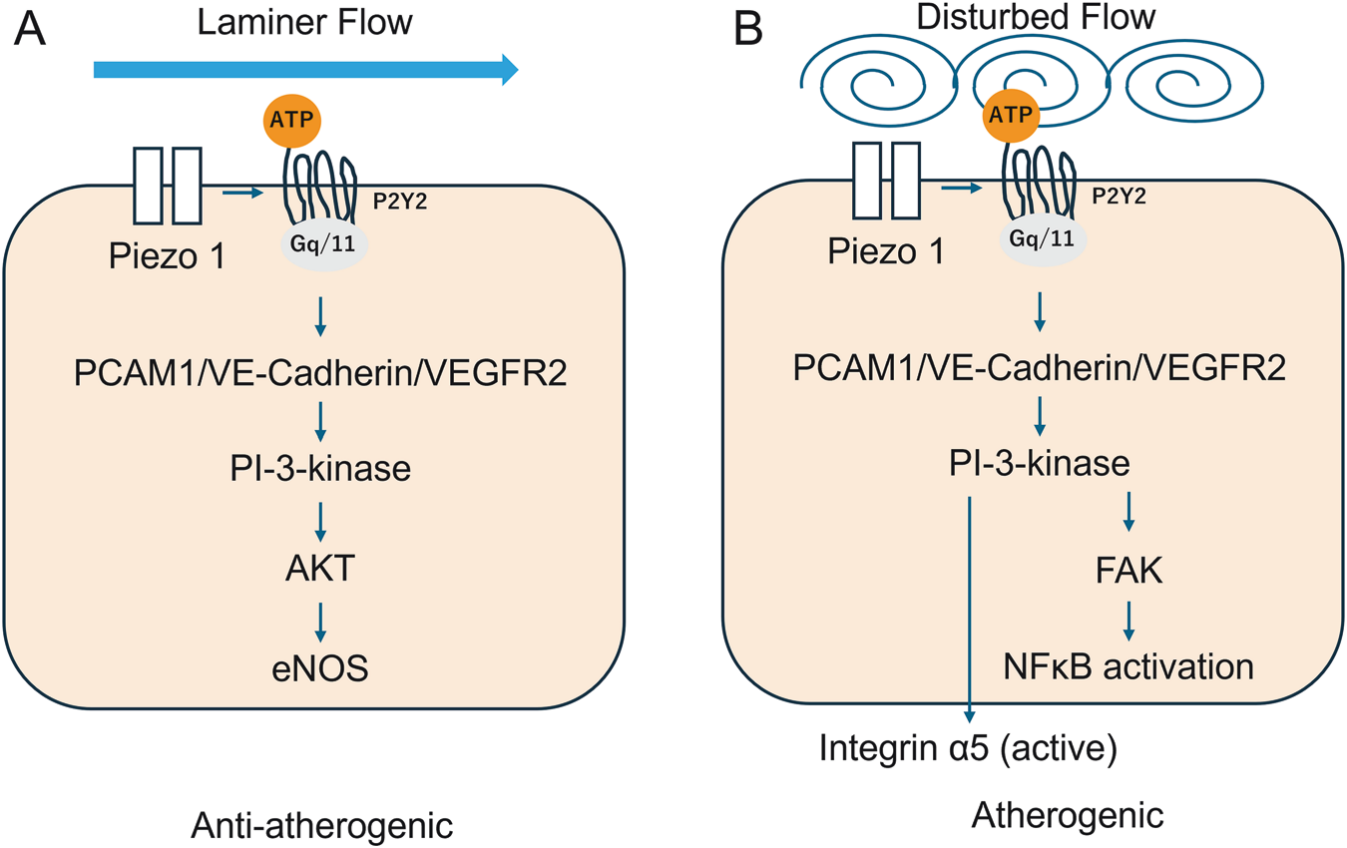
Differential Piezo1 signaling in vascular endothelial cells exposed to laminar versus disturbed flow. Laminar flow **A** and disturbed flow **B** tigger an initial mechanotransduction response that converges on purinergic P2Y2 receptors, G protein alpha subunit Galphaq and G11 (Gq/11) signaling, and the platelet endothelial cell adhesion molecule-1 (PECAM-1)/vascular endothelial cadherin (VE)-cadherin/vascular endothelial growth factor receptor-2 (VEGFR2) complex. Under laminar flow **A**, integrin α5 remains inactive, whereas endothelial nitric oxide synthase(eNOS) becomes active, producing vasodilatory and anti-atherogenic effects. In contrast, disturbed flow **B** engages integrin α5 and nuclear factor kappa-light-chain-enhancer of activated B cell (NF-κB), resulting in a pro-atherogenic transcriptional program

Piezo1 and Piezo2 are mechanosensitive ion channels that play crucial roles in BP control, vasodilation, baroreflex, and renin release [27]. Piezo1, which is expressed in vascular endothelial cells (ECs), senses fluid shear stress and mediates nitric oxide production and vasodilation when blood flow is laminar, acting as an anti-atherogenic signal (Fig. 3) [28, 29]. However, under disturbed flow, Piezo1 can drive inflammatory and atherogenic gene expression via NFκB signaling [30]. Moreover, Piezo1 also contributes to vasoconstriction in mesenteric arteries during exercise, supporting circulatory homeostasis [31]. Both Piezo1 and Piezo2 are indispensable for baroreflex function; their simultaneous deletion in sensory ganglia abolishes reflex bradycardia, resulting in labile hypertension [32]. The broad expression and context-dependent functions of the Piezo channels emphasize their potential as therapeutic targets, although systemic interventions require careful consideration due to possible off-target effects.

## Clinical implications and risk stratification

Integrating new biomarkers and mechanistic insights into clinical practice promises more accurate risk assessments and targeted interventions.

Atrial myopathy, which is characterized by abnormal left atrial (LA) size and function, is associated with an increased risk of atrial fibrillation, heart failure, and dementia [33–35]. Central arterial stiffness, as assessed via cfPWV, is increasingly recognized as a risk factor for atrial myopathy [36]. In a large multicenter cross-sectional study, Mascarenhas et al. revealed that a higher cfPWV was significantly associated with lower LA reservoir and conduit strain, independent of systolic BP and left ventricular morphology and function [37]. Even after excluding participants with hypertension, the association between cfPWV and LA reservoir and conduit strain remained significant. The proposed mechanisms linking arterial stiffness to atrial myopathy include increased cardiac afterload due to premature pulse wave reflections, thereby reducing LA strain. Chronic stress from increased afterload results in LA stretching, stiffening due to fibrosis and myocyte hypertrophy, further impairing strain [38]. The activation of the renin-angiotensin-aldosterone system, augmented by arterial stiffness-related microvascular changes, and impaired coronary perfusion also contribute to the risk of atrial dysfunction [39]. Notably, the present study did not find a significant association between cfPWV and LA contractile strain, possibly because declines in reservoir and conduit strain precede contractile strain impairment. While observational findings suggest a strong association, the mendelian randomization analysis in the atherosclerosis risk in communities study did not provide evidence confirming a causal relationship between genetically predicted arterial stiffness and LA function alterations, potentially due to differences in the measurement methods and study populations. Thus, further prospective studies are needed to establish causality.

Cerebral microbleeds (CMBs) have been associated with hypertensive vasculopathy and significant risks for mortality and disability [40]. A systematic review and meta-analysis confirmed a significant association between higher levels of arterial stiffness, as assessed via PWV, and a greater presence of CMBs [5]. This relationship was observed for both PWVs, with carotid-femoral PWV as a central measurement and brachial-ankle PWV as a peripheral measurement. For central measurements, a PWV > 10 m/s was considered indicative of high risk, whereas for peripheral measurements, a PWV > 18 m/s was considered indicative of high risk [41, 42]. The pooled odds ratio (p-OR) for PWV and CMBs was 1.26 (crude/minimally adjusted) and 1.12 (adjusted for confounders). The underlying mechanism was hypothesized to involve an age-induced reversal of the normal arterial stiffness gradient, wherein central arteries stiffen disproportionately compared with peripheral arteries [43]. This imbalance suggests an increased pulsatile flow transmission and higher flow loads on the susceptible cerebral microvasculature, potentially leading to microvascular damage and structural remodeling [44]. Although this mechanism remains hypothetical, because this systematic review is based on cross-sectional evidence, emerging evidence supports a plausible link between excessive PWV and microvascular injury. Whether PWV can be a useful cardiovascular marker for early detection to delay CMB onset and related neurological consequences remains uncertain and must be validated in prospective observational or interventional studies.

Conventional hypertension management often relies on seated BP measurements, but recent evidence has emphasized supine hypertension as an under-recognized risk factor for cardiovascular events [45]. A multicenter Japanese study revealed that uncontrolled supine hypertension ( ≥ 140/90 mmHg) was significantly associated with an increased risk of cardiovascular and stroke events, independent of seated office BP control [46]. This suggests that supine BP measurements may be closely correlated with prognostically superior nighttime BP. Moreover, the present study reported the synergistic prognostic impact of combining arterial stiffness biomarkers and obstructive vascular biomarkers. Specifically, a higher cardio-ankle vascular index (CAVI) ( ≥ 8.0) and a mildly lower ankle-brachial index (ABI) ( ≤ 1.10) together significantly increased cardiovascular event risk, even in patients with well-controlled office BP. CAVI is a BP-independent measure of arterial stiffness, while ABI is performed to check for peripheral artery disease. These findings suggest that routine evaluation of vascular biomarkers and supine BP may uncover blind spots in current seated BP-based approaches to hypertension management, improved risk stratification and preventive strategies.

Measuring blood pressure in multiple positions is often impractical in routine clinical practice because of time constraints and operational challenges. Accordingly, measuring blood pressure in the supine position is primarily considered in specific situations, such as in patients at high cardiovascular risk, in cases where seated blood pressure is near the diagnostic threshold and classification is uncertain, or when ABPM is not feasible. However, measurement methodology, treatment thresholds, and target levels of supine blood pressure remains insufficiently standardized. Moreover, whether therapeutic intervention for supine hypertension can reduce cardiovascular events is unknown. Therefore, intervention trials are needed to establish the clinical utility of supine blood pressure–guided management.

## Novel assessment methods and biomarkers

The development of non-invasive tools for assessing vascular health is crucial for widespread clinical application and the early detection of diseases. In recent years, several promising methods have been developed.

Endothelial dysfunction is an early stage in the development of atherosclerosis [1]. While ultrasound flow-mediated vasodilation (uFMD) is an established non-invasive method for the assessment of endothelial function, assessments of uFMD require 5-min forearm ischemia in the supine position, a component of the conventional uFMD measurement method that typically takes at least 15 min and demands specialized skills for vessel diameter measurements via ultrasound. A new plethysmographic FMD (pFMD) method has been developed, providing a more convenient way to assess vascular response to reactive hyperemia in the brachial artery. This device uses cuff pressure and volume variations to measure changes in vascular volume. The present study revealed a significant correlation between pFMD and conventional uFMD (β = 0.59, *P* < 0.001) [47]. The mean difference between pFMD and conventional uFMD was 0.78% based on the Bland-Altman plot analysis. The limits of agreement (mean difference ±2 standard deviation of the difference) ranged from -4.53% to 6.11%. The key advantages of pFMD include its fully automated measurement process that requires only a short period of time, making it more convenient and potentially reducing artificial bias. However, the limitations of the present study included validation that was limited to males with low cardiovascular risk profiles and its assessment of volume alterations across the entire upper arm rather than a single brachial artery. Thus, further research on its predictive value for cardiovascular outcomes is needed.

The velocity of waveform propagation in the aorta, along with aortic wall stiffness, has recently been considered a significant predictor of cardiovascular diseases (CVD) risk [41]. The definitive measurement for aortic stiffness is carotid-femoral pulse wave velocity (cfPWV), which reclassifies CVD risk in community-based research when assessed in models that include traditional CVD risk factors [48]. The practical application of aortic stiffness measurements is impeded by the necessity for specific equipment and training essential for the accurate assessment of cfPWV. Gary FM et al. showed that a significant development is the AI-VascularAge (AI-VA) model, a deep learning approach that predicts vascular age from uncalibrated, non-invasive pressure waveforms [49]. Moreover, this model uses a convolutional neural network trained with fixed-scale brachial, radial, and carotid tonometry waveforms to predict negative inverse cfPWV (nicfPWV). The AI-VA which was validated in the Framingham Heart Study showed strong associations with incident CVD (HR, 1.50 [95% CI, 1.24–1.82] per SD; *p* < 0.0001), coronary heart disease (HR, 1.64 [95% CI, 1.24–2.19] per SD; *p* = 0.0006), and heart failure events (HR, 1.65 [95% CI, 1.22–2.23] per SD; *p* = 0.0013), even after adjusting for traditional risk factors. The usefulness of AI-VA is defined by its ability to extract unbiased waveform features agnostically, independent of the calibrated pressure amplitude, heart rate, systolic ejection period, or body size. AI-VA makes it a powerful indicator of vascular health and CVD risk and provides a simple tool for rapid point-of-care assessment, potentially allowing the primordial prevention of cardiometabolic abnormalities associated with accelerated vascular aging. The training and clinical validation cohorts in this study were composed predominantly of White individuals of European ancestry, with limited representation of other racial and ethnic groups. Accordingly, additional validation in more diverse populations is necessary to ensure generalizability and clinical applicability across different demographic backgrounds. Moreover, although the study included a wide adult age range, it did not evaluate performance in children or adolescents. Owing to the substantial difference in physiological characteristics such as body size and heart rate in younger populations, dedicated pediatric and adolescent validation is necessary to confirm applicability in these groups.

The assessment of arterial stiffness has gained importance as a crucial component of cardiovascular risk management, with cfPWV and brachial-ankle pulse wave velocity (baPWV) being widely used measures [48, 50]. However, neither cfPWV nor baPWV can fully capture the stiffness of the proximal aorta, which plays a critical role in the arterial Windkessel function and is particularly susceptible to age-related stiffening. Conversely, heart-to-brachium pulse wave velocity (hbPWV) has emerged as a promising measure that specifically includes the proximal aorta [51, 52]. Sugawara et al. conducted that a large-scale cross-sectional and longitudinal study involving over 7800 Japanese workers and showed that hbPWV was significantly collerated with age (*r* = 0.746–0.796) and Framingham’s general CVD risk score (FRS) (*r* = 0.714–0.749) compared with baPWV (*r* = 0.554 and *r* = 0.643, respectively). Notably, hbPWV maintained its correlation with FRS even after adjusting for age (*r* = 0.260–0.269, *P* < 0.0001) [53]. The study showed a consistent and linear age-related increase in hbPWV from early adulthood, unlike baPWV, which indicated an accelerated increase only after middle age. This suggests that hbPWV may be a more sensitive marker for the early detection of CVD risk. Furthermore, a receiver operating characteristic curve analysis indicated that hbPWV had a more robust ability to stratify general CVD risk (AUC = 0.896–0.913) compared with baPWV (AUC = 0.833). Despite the study’s several limitations, such as the insufficient estimation of the arterial path length and the lack of cfPWV comparisons, its findings emphasized hbPWV’s potential to provide further insights into proximal aortic properties and improve the early detection capabilities of CVD risk, particularly in aging Asian populations.

Pulse pressure amplification (PPA), which is defined as the brachial-to-aortic pulse pressure ratio, is a physiological phenomenon wherein pulse pressure increases as the arterial wave travels from central to peripheral arteries [54, 55]. This amplification naturally decreases with age and is further influenced by cardiovascular risk factors, primarily due to arterial stiffening that causes an earlier return of backward waves [43, 56]. A lower PPA is considered a significant risk factor for adverse cardiovascular outcomes [55]. Huang et al. reported that an individual participant data meta-analysis using the International Database of Central Arterial Properties for Risk Stratification established an outcome-driven PPA threshold of <1.3 as a predictor of cardiovascular and coronary events [57]. In the comparison of PPA < 1.3 with PPA ≥ 1.3, the hazard ratio for cardiovascular events was 1.54 (95% CI, 1.00–2.36), and that for coronary events was 2.45 (95% CI, 1.20–5.01). This threshold specifically identifies individuals at higher risk, particularly those younger than 60 years. The high-risk group (with a PPA threshold of < 1.3) included a significantly higher proportion of women, especially in younger age categories, suggesting that PPA may represent an underestimated risk factor in this demographic. PPA reflects the integration of pulsatile blood pressure (BP), flow, and arterial stiffness gradients, which can result in microvascular injury [58, 59]. Furthermore, these findings support the clinical usefulness of central BP as a routine measurement and emphasize the significance of BP waveform analysis for cardiovascular risk assessment.

## Future perspectives

Future research should focus on validating these novel biomarkers and mechanistic insights using larger, diverse multiethnic cohorts to ensure generalizability. Translating these findings into routine clinical practice will require developing standardized protocols and user-friendly devices. Further investigation of the causal relationships suggested by observational studies, ideally through well-designed prospective trials and advanced genetic analyses, is needed. The primary aim is to translate these advances into individualized care, allowing earlier and more effective therapeutic strategies that result in superior cardiovascular outcomes compared with conventional risk factors alone.

## Conclusion

Historically, vascular function and vascular stiffness were used primarily for physiological characterization and risk association. However, 2025 appears to represent the beginning of a transition in which posture-specific blood pressure, plethysmographic endothelial assessments, and artificial intelligence-derived vascular age are becoming more clinically feasible using emerging implementable devices, and deeper insights into molecular mechanisms and the discovery of new vascular biomarkers continue to refine pathophysiological understanding. Although these tools hold promise for bridging vascular phenotyping and earlier intervention, their incorporation into routine care will still require prospective validation and evidence establishing clinical utility. Collectively, these findings have emphasized the ongoing advancements in the prevention, diagnosis, and management of CVDs.
